# Perspectives of physicians regarding screening patients at risk of hepatocellular carcinoma

**DOI:** 10.1093/gastro/gou089

**Published:** 2015-01-05

**Authors:** Shishira Bharadwaj, Tushar D Gohel

**Affiliations:** Department of Gastroenterology/Hepatology, the Cleveland Clinic Foundation, Cleveland, Ohio, USA

**Keywords:** hepatocellular carcinoma, screening, chronic liver disease, cirrhosis

## Abstract

**Background and aims:** Screening patients at risk for hepatocellular carcinoma (HCC) facilitates early detection of disease, with improved outcome. The most common causes of HCC include chronic viral hepatitis infection—namely hepatitis B, hepatitis C, and cirrhosis. The aim of this study was to assess the awareness of screening among physicians involved in the management of patients at risk for HCC.

**Methods:** Three hundred physicians from three academic centers were invited to participate in a mailed survey questionnaire. The main outcome measure was physicians' knowledge of the current HCC screening guidelines. Demographic and clinical variables were obtained from the survey questionnaire.

**Results:** A total of 177 (59.0%) out of the 300 invited physicians responded to the survey questionnaire, including faculty members (*n =* 129), residents (*n =* 46), and fellows (*n = *2). The specialty areas of the responding physicians were internal medicine (62.1%), family medicine (16.4%), gastroenterology (15.3%), oncology (3.4%) and others (2.8%). The number of physicians who performed HCC screening in patients with cirrhosis secondary to chronic hepatitis B and chronic hepatitis C infection were 163 (92.1%) and 167 (94.4%), respectively; 35.0% of them used alpha-fetoprotein (AFP) every 6 months, while 22.0% used imaging modalities every 6 months to screen for HCC. Further, 22 physicians (12.4%) did not check for serum AFP levels and 33 (18.6%) never used imaging to screen for HCC.

**Conclusion:** The majority of the participating physicians screen high-risk patients for HCC. However, the most appropriate modality of screening (i.e. imaging) is not employed by most physicians and there is greater reliance on AFP levels.

## Introduction

Hepatocellular carcinoma (HCC) is the sixth most prevalent cancer and the third most common cause of cancer-related deaths in the United States [1]. Further, HCC accounts for approximately 90% of all primary liver cancers [2]. The incidence and mortality rates of HCC have been on the rise over the last two decades [2]. The most common predisposing factors for HCC include chronic viral hepatitis B and C infection, alcoholic liver disease and non-alcoholic steatohepatitis [3]. Less-common risk factors include hereditary hemochromatosis, alpha-1 antitrypsin deficiency, primary biliary cirrhosis, autoimmune hepatitis, and Wilson’s disease [4].

Alpha-fetoprotein (AFP) levels and imaging techniques such as ultrasonography are the most common screening modalities used by physicians to detect early HCC [5]. Surveillance of high-risk patients with imaging and/or serum AFP permits early identification and characterization of HCC, resulting in better prognosis [6]. Unfortunately, most cases of HCC are diagnosed in advanced stages, precluding any therapeutic interventions and resulting in decreased median survival rates [7]. The American Association for the Study of Liver Diseases (AASLD) has established guidelines for the surveillance of high-risk patients, including appropriate modalities of screening and the frequency with which such screening methods should be used [8]. For nodules less than 1 cm, the guidelines suggest employing ultrasonography every 3–6 months [8]. In contrast, for nodules larger than 1 cm, the guidelines suggest using computed tomography (CT) scan or magnetic resonance imaging (MRI) [8]. Further, better therapeutic regimens—including liver transplantation for early stage HCC—have also persuaded physicians to screen high-risk patients [9].

In this study, we aimed to assess the awareness of HCC screening among physicians involved in the care of high-risk patients. We also aimed to assess their knowledge of the appropriate screening strategies and the frequency of such strategies.

## Materials and methods

### Participants and process

Between June and August 2012, 300 physicians, including residents and fellows at three academic centers (Cleveland Clinic, Metrohealth Hospital and Fairview Hospital) were randomly invited to participate in a survey questionnaire, which was prepared and mailed to physicians using Research Electronic Data Capture (REDcap), a secure web application.

### Survey questionnaire

A survey questionnaire relevant to HCC screening was used. Since there is lack of validated questionnaires in the literature, we included questions pertaining to the choice of screening modality and also the frequency of using such modalities, based on the AASLD guidelines. Demographic data including age, gender, area of specialty, and level of training (faculty, fellow, or resident) was also incorporated in the questionnaire. Additionally, the survey included questions about HCC screening, including risk groups screened for HCC, the screening test (AFP *vs*.** imaging), frequency of screening, immunization history, HIV status, vaccination history and physician responsibility.

### Outcome measurement

The primary outcome was a measurement of awareness among physicians of available choices of screening modality and the frequency of use of such modalities, based on AASLD guidelines for HCC.

### Statistical analysis

Descriptive statistics were computed for all variables. The responses were analysed and each answer was represented as a proportion of the physicians who responded. The percentage of physicians employing each screening test was determined separately for AFP and imaging.

## Results

One hundred and seventy-seven physicians (59.0%) responded to the survey questionnaire, of which 126 (71.2%) were male; the majority (79.7%) were below 50 years of age. The physician group included faculty members (*n = *129), residents (*n = *46), and fellows (*n = *2). Their specialty areas included internal medicine (62.1%), family medicine (16.4%), gastroenterology (15.3%), oncology (3.4%) and others (2.8%) (Table 1).

**Table 1.** Characteristics of physicians who responded to the survey (*n = *177)

Characteristics	Number
Gender	
Male	126 (71.2%)
Female	51 (28.8%)
Age range	
22–35	66 (37.3%)
36–50	75 (42.4%)
51–65	32 (18.1%)
>65	4 (2.3%)
Specialty	
Internal medicine	110 (62.1%)
Family medicine	29 (16.4%)
Gastroenterology	27 (15.3%)
Oncology	6 (3.4%)
Others	5 (2.3%)*
Level of training	
Faculty members	129 (72.9%)
Residents	46 (26.0%)
Fellows	2 (1.1%)

*One person did not respond.

The majority of the physicians performed HCC screening on high-risk patients including those with chronic hepatitis C with cirrhosis (94.4%), chronic hepatitis B with cirrhosis (92.1%) and alcoholic liver disease (75.7%). Also, HCC screening was performed on patients diagnosed with hereditary hemochromatosis with underlying cirrhosis (80.8%), primary biliary cirrhosis (67.8%), chronic hepatitis B without cirrhosis (52.0%), autoimmune hepatitis (31.1%), and in patients with a history of colon cancer (16.4%) (Table 2).

**Table 2.** Proportion of physicians screening each patient group (*n = *177)

Diagnosis	Number		
	Yes	No	Uncertain
Chronic hepatitis B carriers without cirrhosis	92 (52.0%)	80 (45.2%)	5 (2.8%)
Chronic hepatitis B patients with cirrhosis	163 (92.1%)	12 (6.8%)	2 (1.1%)
Chronic hepatitis C patients with cirrhosis	167 (94.4%)	8 (4.5%)	2 (1.1%)
Past history of colon carcinoma	29 (16.4%)	145 (81.9%)	3 (1.7%)
Alcoholic liver cirrhosis	134 (75.7%)	39 (22.0%)	4 (2.3%)
Genetic hemochromatosis with cirrhosis	143 (80.8%)	32 (18.1%)	2 (1.1%)
Primary biliary cirrhosis	120 (67.8%)	51 (28.8%)	6 (3.4%)
Autoimmune hepatitis	55 (31.1%)	115 (65.0%)	7 (4.0%)

Sixty-two physicians (35.0%) used 6-monthly AFP levels to screen for HCC, while 77 (43.5%) used AFP levels every 9 months. Thirty-nine physicians (22.0%) used imaging every 6 months and 86 (48.6%) used imaging every 9 months. Further, 22 (12.4%) physicians did not check for serum AFP levels and 33 (18.6%) never used imaging to screen for HCC (Table 3).

**Table 3.** Screening interval for alpha-fetoprotein (AFP) and imaging modalities (*n = *177)

Interval for screening	Number	
	AFP method	Imaging method
Never	22 (12.4%)	33 (18.6%)
Every 3 months	7 (4.0%)	2 (1.1%)
Every 6 months	62 (35.0%)	39 (22.0%)
Every 9 months	77 (43.5%)	86 (48.6%)
Used method other than AFP/Imaging	9 (5.1%)	17 (9.6%)

AFP = alpha-fetoprotein

Additionally 41.2% of the physicians responded that the screening of at-risk patients for HCC should be the combined responsibility of gastroenterologists and primary care physicians (Table 4). Also, 31.1% and 24.8% responded that responsibility for HCC screening rested with gastroenterologists and primary care physicians, respectively. Only 2.8% of the physicians responded that oncologists should take on responsibility for screening for HCC.

**Table 4.** Responsibility to screen high-risk patients (*n = *177)

Specialty that should take the responsibility	Number
Shared care between gastroenterologists and family physicians	73 (41.2%)
Gastroenterologists	55 (31.1%)
Family physicians	44 (24.8%)
Oncologists	5 (2.8%)
General surgeons	0%
Others	

## Discussion

Our study was designed to investigate physicians' awareness of HCC screening. We found that, although the majority did screen high-risk groups for HCC, most did not employ the appropriate screening strategy and its frequency of use, as established by the AASLD.

The majority of HCCs are diagnosed in advanced stages, which carries a poor prognosis [10]. A striking difference is noted in the survival rates of patients with early or limited HCC, who are likely to be cured or may benefit from a greater disease-free interval when diagnosed early [11]. Screening aims at decreasing the incidence of mortality caused by a specific 3disease [11]. The slow and insidious nature of HCC and the survival benefit associated with early detection makes screening an effective strategy [12]. It is recommended that at-risk patients be screened with an HCC incidence of >1.5% per year for the screening strategy to be cost-effective [12]. Chronic hepatitis C infection with cirrhosis is now the leading risk factor for HCC in the United States and is responsible for the recent increase in the incidence of HCC [4]. Also, the annual incidence of HCC in patients with less-common risk factors—such as hemochromatosis (especially with established cirrhosis), alpha 1 anti-trypsin deficiency and primary biliary cirrhosis (stage 4)—was shown to be >1.5%, warranting the screening of such patients [13–16]. In our study, we found that the majority of the participating physicians screened high-risk patients such as those with chronic hepatitis C with cirrhosis, chronic hepatitis B with cirrhosis and cirrhosis due to alcoholic liver disease. However, fewer screened patients with underlying hereditary hemochromatosis, primary biliary cirrhosis, or chronic hepatitis B without cirrhosis. Our study did not include non-alcoholic steatohepatitis, which is under investigation as one of the risk factors for HCC. However, the evidence is indirect and the risk–effect association has not been established yet [17].

This study also showed that a greater proportion of physicians screened patients at risk for developing HCC every 9 months (43.5% using AFP levels and 48.6% with imaging studies) than those who screened every 6 months (35.0% with AFP levels and 22.0% used imaging modalities). Although there is a lack of evidence regarding the benefit of 6-monthly surveillance over 9 monthly, the AASLD recommends that patients at risk for HCC should be screened every 6 months [8]. The proportion of physicians relying on AFP levels for screening purposes was higher than those using imaging. Ultrasonography as a screening test has a sensitivity of 65–80% and specificity of more than 90%—while AFP has sensitivity of 66% and specificity of 82%—and is the test recommended by the AASLD [18, 19]. Although our study did investigate the relative screening frequencies of AFP and imaging modalities used by physicians, we did not assess the type of screening modality most commonly employed by the majority. This hinders drawing any conclusions regarding the screening strategy used by physicians in our study.

Our study also showed that the majority of physicians thought that screening of high-risk patients should be the responsibility of gastroenterologists and primary care physicians, when compared to either alone. A study conducted by Sharma *et al*.** showed that 79% of the gastroenterologists identified high-risk patients, among whom 88.5% and 98% were aware of the appropriate screening strategy and frequency of its use [20]. Our study further supports these findings. However, the physicians who responded to our survey belonged to diverse specialties, strengthening the internal validity of our study.

There are several limitations to our study. Firstly, it may have suffered from significant selection bias, as the participating physicians may not be representative of the entire physician population who screen high-risk groups for HCC. Secondly, we did not categorize the fellows and residents according to their level of training, which could have further biased our results. Thirdly, we did not use a validated survey questionnaire, owing to the non-existence of such an instrument in HCC screening. Fourthly, the responses may also have been subject to recall bias. Finally, we did not differentiate between the different imaging modalities available. However, we believe that our study results would lead to future research avenues to create a validated survey questionnaire for HCC screening and mitigate the knowledge gap among physicians who are involved in the care of HCC patients.

In conclusion, the majority of the physicians screened patients who were at high risk of developing HCC. However, less-common risk groups were not routinely screened and physicians should be made aware of such discrepancies in their screening strategies. Moreover a considerable number of physicians were unclear about available screening modalities and the frequency of use. There are no validated quality assessment tools to measure the adequacy of screening HCC among at-risk patients. Developing such quality indicators would enable us to screen for early HCC, increase disease-free survival among such patients and decrease the cost burden.

*Conflict of interest statement:* none declared.
